# Patient Reported Outcomes Measurements Information System in Stroke Patients in Full and Shortened Format

**DOI:** 10.3389/fneur.2020.630850

**Published:** 2021-01-22

**Authors:** Charlotte Lens, Jelle Demeestere, Kris Vanhaecht, Robin Lemmens

**Affiliations:** ^1^Department of Neurology, University Hospitals Leuven, Leuven, Belgium; ^2^Department of Neurosciences, Experimental Neurology, KU Leuven – University of Leuven, Leuven, Belgium; ^3^Center for Brain & Disease Research, Laboratory of Neurobiology, VIB, Leuven, Belgium; ^4^Leuven Institute for Healthcare Policy - Department of Public Health, KU Leuven - University of Leuven, Leuven, Belgium; ^5^Department of Quality Management, University Hospitals Leuven, Leuven, Belgium

**Keywords:** stroke, PROMs, PROMIS, health domains, validation

## Abstract

**Introduction:** The modified Rankin Scale (mRS) after 90 days documents outcome in stroke patients, but focusses only on activities of daily living. Here we studied stroke outcome beyond these activities by the Dutch-Flemish version of the Patient Reported Outcomes Measurement Information System (PROMIS) questionnaire.

**Patients and Methods:** We documented the mRS at day 90 in stroke patients who filled out a questionnaire on pain intensity and seven PROMIS domains: physical function, ability to participate in social roles, anxiety, fatigue, depression, sleep disturbance, pain interference. In a subgroup of patients this questionnaire was reduced to one overall question per PROMIS domain. We correlated these findings with the mRS.

**Results:** We received 102 questionnaires and identified physical function as the most affected PROMIS domain. The strongest correlation with mRS was found for the health domains of physical function (ρs = 0.70, *p* < 0.001) and ability to participate in social roles (ρs = 0.61, *p* < 0.001). The other domains with substantial proportions of patients with worse scores compared to the general population (19–44%) correlated weakly with the mRS. We identified a strong correlation between the single question per health domain and the overall score per PROMIS domain.

**Discussion and Conclusion:** PROMIS better reflects the overall health status of stroke patients beyond functional outcome as measured by the mRS. Simplification of the questionnaire with a single question per PROMIS domain could potentially replace the full questionnaire, but needs further validation.

## Introduction

Stroke affects several health domains which may lead to disability and disturbs the life of patients and their family. Stroke outcome is conventionally measured by assessing disability. Developed tools quantify deficits that clinicians consider important, but these tools may not cover the full range of concerns and symptoms that patients experience (1). The modified Rankin Scale (mRS) is the most commonly used functional outcome measure in stroke studies. This instrument measures the functional disability of the patients using an ordinal hierarchical scale with scores ranging from zero (no symptoms) to six (death) (2–4). However, the scale neglects many other domains like mood, global cognitive function, pain, fatigue, feeding, social roles, self-care, and communication (2, 5). In addition, some heterogeneity exists in the scoring systems for various outcome measurements currently used in clinical trials e.g., the mRS, Barthel index, and National Institutes of Health Stroke Scale since they assess different symptoms and consequences of stroke (3). This can complicate the interpretation of test results and comparison between populations. Patient-Reported Outcome Measurements (PROMs) may provide an attractive alternative for all these problems by a patient-oriented assessment of health status and quality of life (2). The Patient-Reported Outcome Measurement Information System (PROMIS) can efficiently assess different health domains using a continuous scale and intends to quantify health status in individuals with a wide range of symptoms and diseases. The results can be compared to the general population enabling identification of typical clusters of affected health domains for a specific disease (2). Specifically in stroke patients knowledge on a broader spectrum of health concerns and symptoms can aid the organization of rehabilitation programs (6). Previously a study selected and reported on six PROMIS domains in patients after stroke in the United States. They found that these patients, reported symptoms in multiple health domains, which are not always captured by only using the mRS (2).

We aimed to assess the feasibility of the Dutch-Flemish version of the PROMIS-questionnaire in stroke previously reported on in the United States. Furthermore, we investigated the proportion of stroke patients with meaningfully different scores from the general population in these various domains and correlated patient reported outcomes with clinician-reported outcome (the mRS). In addition, in a subgroup we examined the value of replacing the questionnaire with a single question per PROMIS-domain in order to decrease the time and burden for patients in filling out these forms.

## Methods and Materials

### Design, Setting, and Patients

We performed a prospective, cross-sectional study of stroke patients in an outpatient clinic 3 months after their index event at the University Hospitals Leuven from December 20th, 2018 to April 18th, 2019. The study was approved by the Research Ethics Committee UZ/KU Leuven. Patients were eligible if they were 18 years or older, were previously diagnosed with ischemic or hemorrhagic stroke and if they visited the outpatient clinic 3 months after the admission for stroke. We collected PROMs using a paper version of the PROMIS v2.1-Profile-57 questionnaire. Patients received the questionnaire during the visit to the outpatient clinic after signing an informed consent form. Patients completed the questionnaire at home with the help of a proxy if needed and returned the questionnaire by mail.

### Outcome Measurements

We assessed eight health domains: pain intensity according to the numeric rating scale (NRS-11) and seven PROMIS scales: physical function, ability to participate in social roles, fatigue, anxiety, depression, pain interference, and sleep disturbance. Since we included the PROMIS domain of pain interference we also decided to assess pain intensity via an NRS-11 pain score. The aim of the study was to assess the feasibility of the Dutch-Flemish version of the PROMIS-questionnaire. Unfortunately, the lack of a Dutch translation of the PROMIS tool to determine Quality of life (Qol) or the Neuro-Qol hampered including these assessments in our study. In the second half of the study we added one comprehensive question, regarding each PROMIS health domain, according the principle of the NRS-11, which means that the patient indicates the health status for each PROMIS health domains on a scale from 0 to 10 (2).

Clinicians completed and recorded the mRS score during the outpatient visit before patients filled out the questionnaires and were therefore blinded for the results of the PROMIS questionnaires. The mRS measures the global disability of patients with scores ranging from 0 (no symptoms) to 6 (death). After completion of the questionnaire we contacted all patients by phone to examine the feasibility of filling out the PROMIS-questionnaire. We considered the time needed to complete the questionnaire, the need for assistance and whether patients and their proxy could easily understand the questions.

### Statistical Analysis

The scores on the 7 PROMIS health domains were compared to the general population using response pattern scoring, using the scoring service of The Assessment Center software (7). The assessment center is an online tool for data collection that facilitates researchers to create study-specific websites for capturing participant data securely online. Such a tool was developed for PROMIS-questionnaires to convert the answers on the questions to T-scores for the different health domains. T-scores were obtained and transformed, with higher scores indicating worse health. The mean in the general population is set at 50 and a meaningful difference from this population mean, defined as an increase in the T-score ≥5 points, indicates worse health (6). We calculated mean T-scores per PROMIS health domain, the proportion of the T-scores per PROMIS health domain that were meaningfully different from the general population and the mean T-score for each mRS category. Results of the NRS-11 questionnaire for pain assessment were also transformed, with higher scores indicating worse health. Qualitative data regarding questionnaire self-administration were analyzed using descriptive statistics. We determined Spearman correlation coefficients between the mRS and the need of assistance of filling out the questionnaire; the results of the PROMIS questionnaires and the mRS; the PROMIS-questionnaires and the one comprehensive question; and the mRS and the one comprehensive questionnaire. Statistical significance was set as a *p* < 0.05. We used Statistica version 13.5.0.17 © 2017 TIBCO Software Inc. for all analyses.

## Results

### Patient Characteristics

**Figure 1:**
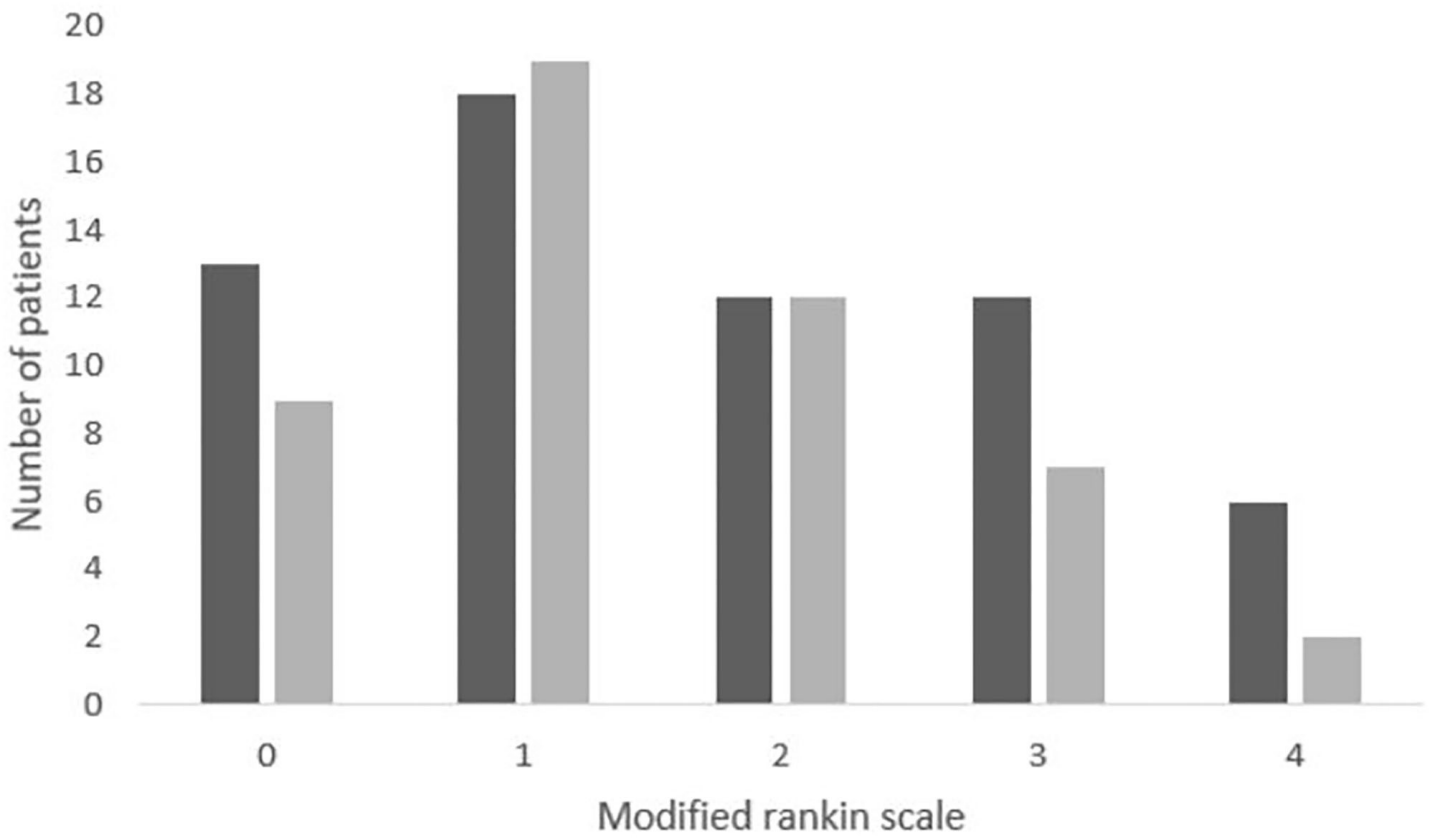
Distribution of the mRS in the patient population. The number of patients in each mRS category. We included 22 patients with a mRS score of 0 (18.8%), 41 with a mRS score of 1 (35.0%), 24 with a mRS score of 2 (20.5%), 21 with a mRS score of 3 (17.9%), and 9 with a mRS score of 4 (9.9%). The shaded proportion of the columns bars indicate the patient who completed both the PROMIS questionnaire and the NRS-11 questionnaire.

During the study period, 143 patients who fulfilled the inclusion criteria presented in the outpatient clinic 3 months after the index stroke. Of those 119 signed the informed consent form and received a questionnaire. At the end of the study period, 102 patients returned the completed questionnaire corresponding to a response rate of 85.7%. The median mRS in this cohort was 1 (IQR 1–3). In 49 of the 102 patients (48%) the comprehensive question, regarding each health domain, according the principle of the NRS-11, was added (Figure 1). Patients with a mRS score of 5 or 6 were not included in this study since they did not present at the outpatient clinic.

We could evaluate the need for assistance during questionnaire completion in 92 patients. Caretakers provided assistance in 22 patients (24%) and 11 patients (12%) expressed difficulties understanding the questions. The need for assistance or inability to fill out the questionnaire moderately correlated with the mRS score (ρs = 0.44, *p* < 0.001). None of the patients with a mRS score of 0 needed assistance, as opposed to 6 patients (18%) with mRS score 1; 5 with an mRS score of 2 (22.7%); 7 with mRS score of 3 (53.8%); and 4 with a mRS score of 4 (80.0%). The median time a patient needed to complete the questionnaire was 20 min (IQR 12–30 min).

### Ranking of the PROMIS-Domains According to the Mean T-Score

The most affected health domains after stroke were physical function and ability to participate in social roles. The domain of sleep disturbance was the least influenced by the index stroke (Table 1).

**Table 1:** Ranking of the PROMIS-domains according to the mean T-score.

**PROMIS health domain**	**Number of patients**	**Mean ± SD**	**Scores meaningfully worse than general population, *n* (%)**
Physical function	102	57.43 ± 2.75	83 (81,4)
Ability to participate in social roles	101	51.52 ± 2.28	74 (72,6)
Anxiety	102	52.46 ± 2.97	44 (43.1)
Depression	102	50.72 ± 3.27	37 (36.3)
Fatigue	102	50.26 ± 2.27	31 (30.4)
Pain interference	100	50.18 ± 3.66	33 (33.0)
Sleep disturbance	101	48.91 ± 2.79	19 (18.6)

### Correlation Between PROMIS and mRS

**Figure 2:**
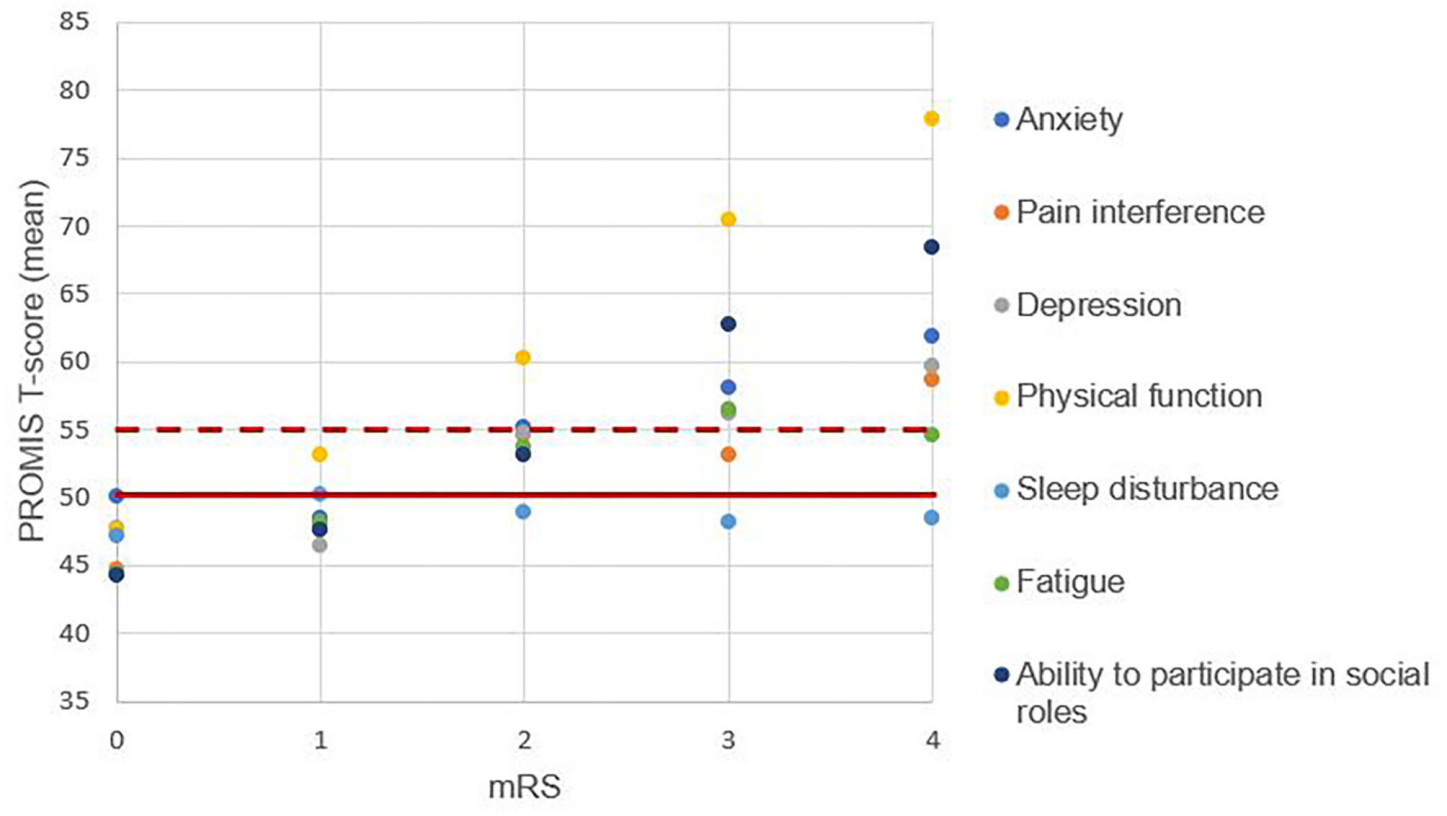
Patient-reported outcomes across the levels of disability. One dot represents the mean T-score. The solid horizontal line represents the mean of the general population, the dotted horizontal line represents the score that is meaningfully worse than the general population. A higher T-score represents worse patient-reported health.

We found no differences between PROMIS scores of patients with mRS of 0 and the general population for all health domains. We observed similar findings in patients with a mRS of 1 except for the domain of physical function (53.12 ± 2.88, *p* = 0.05). Patients with a mRS score of ≥2 had worse T-scores compared to the general population for all health domains, except for the domain of sleep disturbance (Figure 2 and Supplementary Figures 1–7).

A correlation between the health domains and the mRS could be demonstrated for seven of the eight patient-reported health domains. We found no correlation for the domain of sleep disturbances (*p* = 0.94). The strongest correlation with the mRS was found for the health domains of physical function (ρs = 0.70, *p* < 0.001) and ability to participate in social roles (ρs = 0.61, *p* < 0.001). The domains fatigue (ρs = 0.37, *p* < 0.001), pain interference (ρs = 0.36, *p* < 0.001), depression (ρs = 0.34, *p* < 0.001), pain intensity (ρs = 0.32, *p* = 0.001), and anxiety (ρs = 0.32, *p* = 0.001) correlated rather weakly with the mRS.

### Comprehensive Question Regarding Each Health Domain

**Figure 3:**
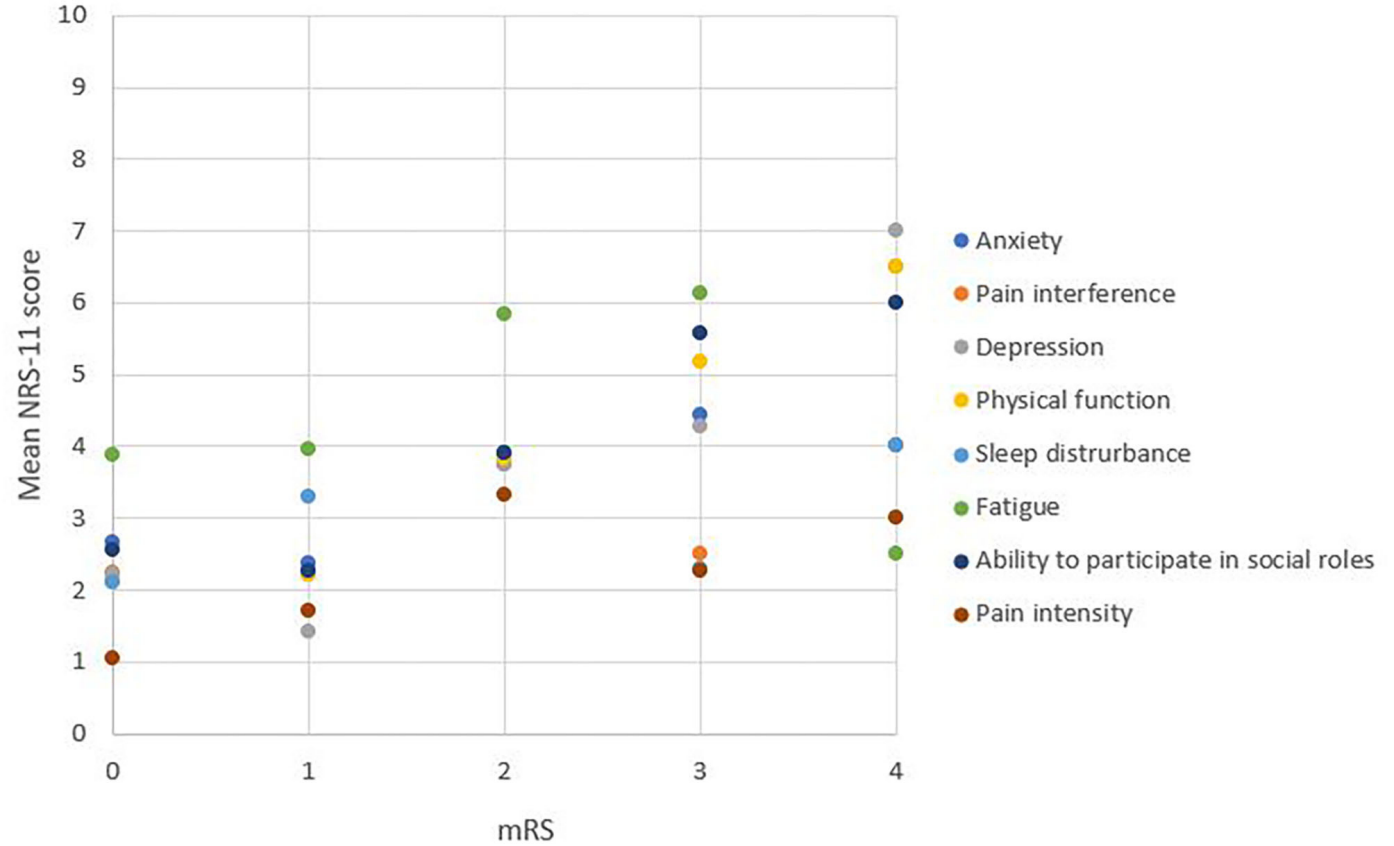
NRS-11 scale of each health domain across the levels of disability. One dot represents the mean score on the scale of zero to ten. A higher NRS-11 score represents worse patient-reported health.

In half of the study population we additionally assessed the health domains based on one comprehensive question (Figure 3 and Supplementary Figures 8–15). The results of the comprehensive questionnaires correlated with the results of the PROMIS questions, in descending order: pain interference, ρs = 0.87 (*p* < 0.001); anxiety, ρs = 0.83 (*p* < 0.001); depression, ρs = 0.82 (*p* < 0.001); ability to participate in social roles, ρs = 0.76 (*p* < 0.001); physical function, ρs = 0.71 (*p* < 0.001); sleep disturbance, ρs = 0.71 (*p* < 0.001); and fatigue, ρs = 0.60 (*p* < 0.001).

The relationship between the score on the comprehensive question and the mRS score was studied similarly as for the PROMIS questions. The domains with the strongest correlation were depression (ρs = 0.48; *p* < 0.001), ability to participate in social roles (ρs = 0.47; *p* < 0.001) and physical function (ρs = 0.46; *p* < 0.001), followed by pain interference (ρs = 0.32; *p* = 0.03), and anxiety (ρs = 0.29; *p* = 0.05). There was no correlation with the domains of sleep disturbance (*p* = 0.53) and fatigue (*p* = 0.08).

## Discussion

In this study we showed that most PROMIS health domains were affected in stroke patients who were followed-up at the outpatient clinic 90 days after stroke admission. We also identified a correlation between the mRS and PROMIS health domains. But the strength of the correlations varied by domain, suggesting that not all health domains are fully captured by the mRS. Replacing the PROMIS-questionnaires with one comprehensive question strongly correlated with the overall score per domain based on the full questionnaire and resulted in similar correlations with the mRS. Although previously strongly correlated domains show a possibly relevant decrease in correlation strength which could be the result of limiting the domain to one single question.

In this study, the health domain of physical function was most affected in stroke patients, followed by the domain of ability to participate in social roles. Since stroke is the most important cause of physical disability in adults, physical function is already the primary focus of most rehabilitation programs (2, 8). However, stroke care and rehabilitation programs have focused less on social participation, notwithstanding that interventions on improving social participation are successful in stroke patients, since they avoid depression and worsening of function, quality of life, and health status (2, 9). The domains anxiety, depression, fatigue, and pain interference were moderately affected in patients with stroke. These results are in accordance with previous studies (10–14). By assessing all health domains in stroke patients, rehabilitation programs can be tailored to individual health deficits which require the most attention (2). Sleep disturbances was the only health domain for which the patients in this sample did not report worse scores compared to the general population. Although more than 50% of patients with stroke have sleep-disordered breathing (e.g., obstructive sleep apnea), these sleep disorders often remain unnoticed by the patient, which may explain the lack of identification of sleep disturbances in stroke patients in this and other studies (2, 15).

We evaluated the PROMIS health domains across levels of disability, represented by the mRS score. Patients with a higher level of disability (mRS 2, 3, or 4) had an increasing T-score for all health domains, except for sleep disturbances, although correlation strength varied for different domains (16, 17). As expected based on previous research (2, 6, 16), the PROMIS domain of physical function showed the strongest correlation with the mRS. This is not surprising since the mRS mainly assesses motor function (2). PROMIS health domains less reliant on motor function showed a lower correlation with the mRS. The variability of correlation strength suggests that the mRS fails to assess the complete health status, and non-motor disability may be insufficiently captured by the mRS. Therefore, the sole use of the mRS as outcome measurement may limit insights in various health domains that contribute to the quality of life.

We found a strong correlation between each single comprehensive question per PROMIS health domain and the overall score of the PROMIS health domain questionnaire. This suggests that one comprehensive question per health domain can collect similar information compared the eight PROMIS-questions per domain. However, in contrast to PROMIS, this single question has not been studied in a control group of healthy volunteers and therefore values in the normal population are lacking. A limitation of this substudy was the relatively low sample size of only 49 patients and therefore this needs further validation. The validation of the single question per PROMIS domain in other and larger populations, e.g., a healthy population, and settings can be the objective for further investigation.

This study has several strengths. Study participation was discussed with all stroke patients during routine care. This increases the generalizability of the findings compared to outcome variables exclusively obtained in clinical studies (2). The availability of the mRS score made it possible to establish the correlation between the patient and the clinician reported outcomes (6, 18, 19). The high response rate and the low percentage of patients who needed assistance or could not understand the questions reflect the feasibility of obtaining PROMIS-questionnaires in this patient population. Finally, the results of this study are likely valid since they are in line with results of previous studies in both small and large sample sizes (2, 6). The newly developed single question per domain in this study could be an interesting tool for future studies since this might improve the feasibility of obtaining PROMs. There are also limitations. First, only eight health domains were investigated during this study. Other domains, like mood, quality of life, cognitive functioning, and ability to communicate, are also affected after stroke, but were beyond the scope of this study (5). Second, only patients seen at the outpatient clinic could participate. Information on patients with severe functional disability (mRS score >4) is therefore lacking. However, severely disabled patients require constant attention and nursing care and are often not able to answer the questionnaire. In addition, patients with cognitive impairments or aphasia were excluded from this study since they would not be able to fill out the questionnaire even with assistance. Thirdly, other studies have already demonstrated that other characteristics, like age, sex, social background, cognition, and mood are independently associated with patients-reported outcomes after stroke. We did not collect these variables in this study and adjustments for these variables was therefore not possible (2, 20, 21). Fourthly, the shortened questionnaire with one question for each health domain was added at the end of the PROMIS-questionnaire which could have introduced bias. More specifically, the process of reflection over the previous questions may have influenced the rating of this general score. Lastly, one out of four patients received help to answer the questions. This may have increased the T-scores since proxy's tend to report worse patient-reported outcomes compared to the patient (20, 22).

In conclusion obtaining PROMIS during stroke patient care is feasible and provides additional outcome information. Patients with stroke had worse outcomes in comparison with the general population for all domains, except for sleep disturbance. Physical functioning and ability to participate in social roles were the most affected domains in this population. Currently only a limited amount of attention is given to other affected domains like depression and anxiety possibly since it is not sufficiently captured by the mRS. Stroke clinicians should try to obtain PROMIS results in their patients to enable individualized based care during follow-up which covers all the affected health domains. Shortening of this questionnaire to only one question per domain could be the aim of future studies.

## Data Availability Statement

The raw data supporting the conclusions of this article will be made available by the authors, without undue reservation.

## Ethics Statement

The studies involving human participants were reviewed and approved by Research Ethics Committee UZ/KU Leuven. The patients/participants provided their written informed consent to participate in this study.

## Author Contributions

CL, KV, and RL: conception and design of study, analysis, and/or interpretation of data. CL: acquisition of data and drafting the manuscript. JD, KV, and RL: revising the manuscript critically for important intellectual content. CL, JD, KV, and RL: approval of the version of the manuscript to be published. All authors contributed to the article and approved the submitted version.

## Conflict of Interest

The authors declare that the research was conducted in the absence of any commercial or financial relationships that could be construed as a potential conflict of interest.
