# Supplementary material for: Patient Reported Outcomes Measurements Information System in Stroke Patients in Full and Shortened Format
Source: Front Neurol. 2021 Jan 22;11:630850. doi: 10.3389/fneur.2020.630850 (PMC7864084; doi:10.3389/fneur.2020.630850)
Supplement: Supplementary file 1 [file Data_Sheet_1.pdf]

frontiers

Supplementary Material

1 Supplementary Figures and Tables

1.1 Supplementary Figures

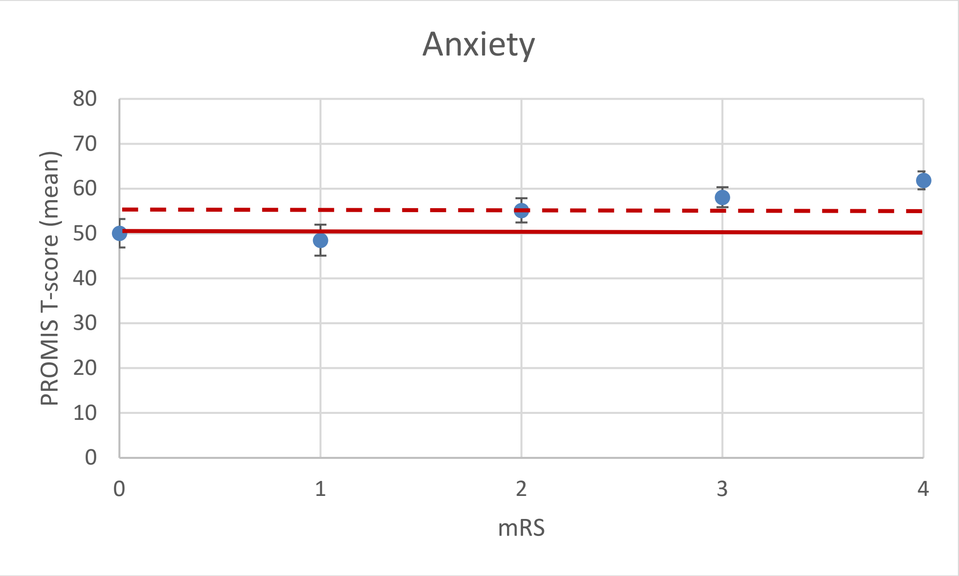

Supplementary figure 1 One dot represents the mean T-score; whiskers are the standard error of the T-score. The T-scores for mRS 0, 1, 2, 3 and 4 are respectively: 50.06 ± 3.17; 48.52 ± 3.45; 55.16 ± 2.70; 58,07 ± 2.23; 61,84 ± 2.00. The solid horizontal line represents the mean of the general population, the dotted horizontal line represents the score that is meaningfully worse than the general population. A higher T-score represents worse patient-reported health.

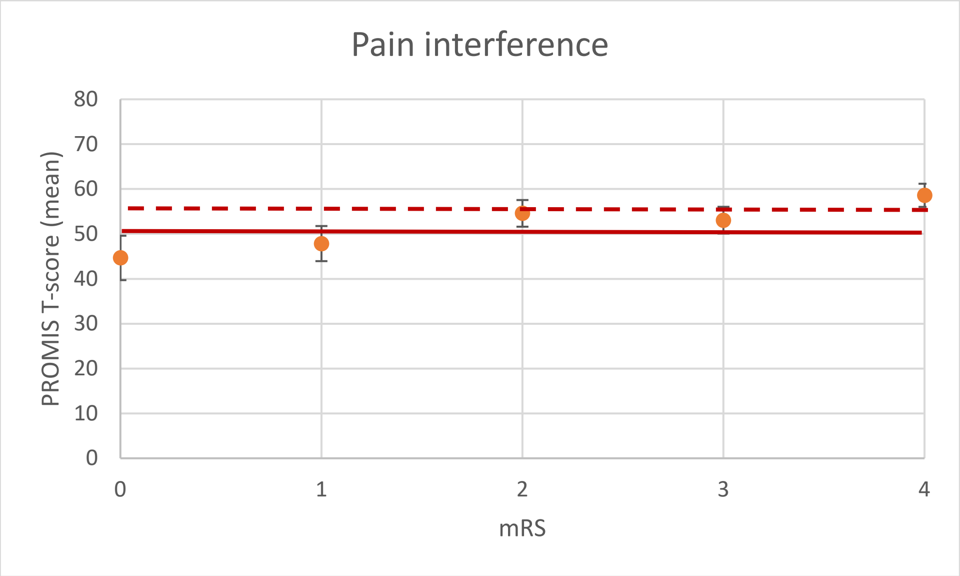

Supplementary figure 2 One dot represents the mean T-score; whiskers are the standard error of the T-score. The T-scores for mRS 0, 1, 2, 3 and 4 are respectively: 44.65 ± 4.95; 47.84 ± 3.91; 54.58 ± 2.97; 53.06 ± 2.98; 58.60 ± 2.58. The solid horizontal line represents the mean of the general population, the dotted horizontal line represents the score that is meaningfully worse than the general population. A higher T-score represents worse patient-reported health.

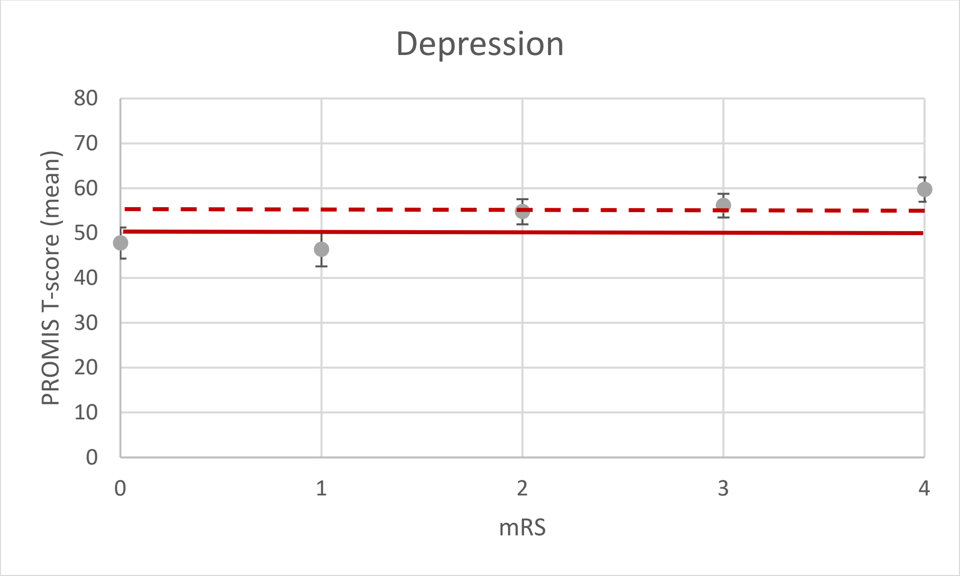

Supplementary figure 3 One dot represents the mean T-score; whiskers are the standard error of the T-score. The T-scores for mRS 0, 1, 2, 3 and 4 are respectively: 47.78 ± 3.46; 46.38 ± 3.81; 54.75 ± 2.80; 56.11 ± 2.64; 59.7 ± 2.72. The solid horizontal line represents the mean of the general population, the dotted horizontal line represents the score that is meaningfully worse than the general population. A higher T-score represents worse patient-reported health.

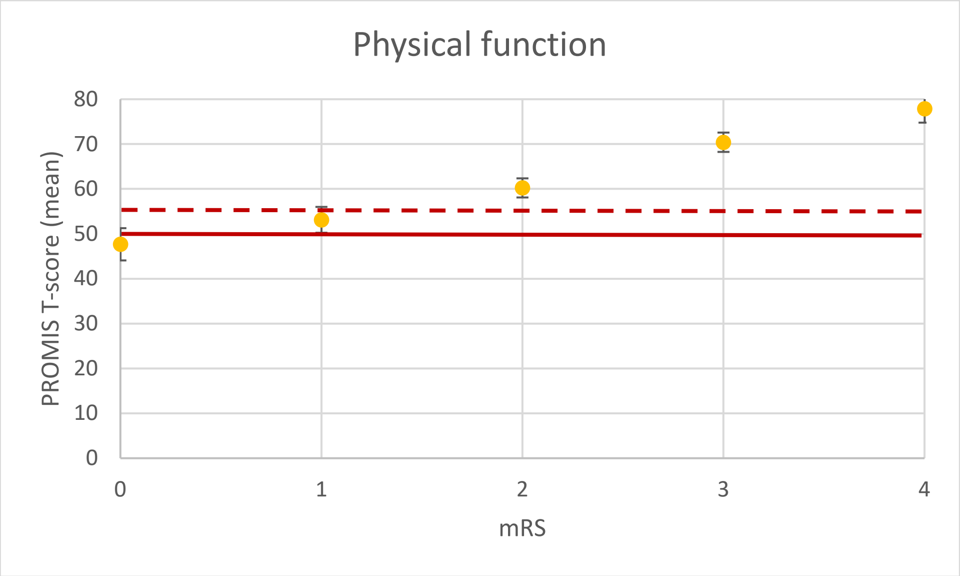

Supplementary figure 4 One dot represents the mean T-score; whiskers are the standard error of the T-score. The T-scores for mRS 0, 1, 2, 3 and 4 are respectively: 47.67 ± 3.60; 53.12 ± 2.88; 60.24 ± 2.13; 70.41 ± 2.15; 77.86 ± 3.08. The solid horizontal line represents the mean of the general population, the dotted horizontal line represents the score that is meaningfully worse than the general population. A higher T-score represents worse patient-reported health.

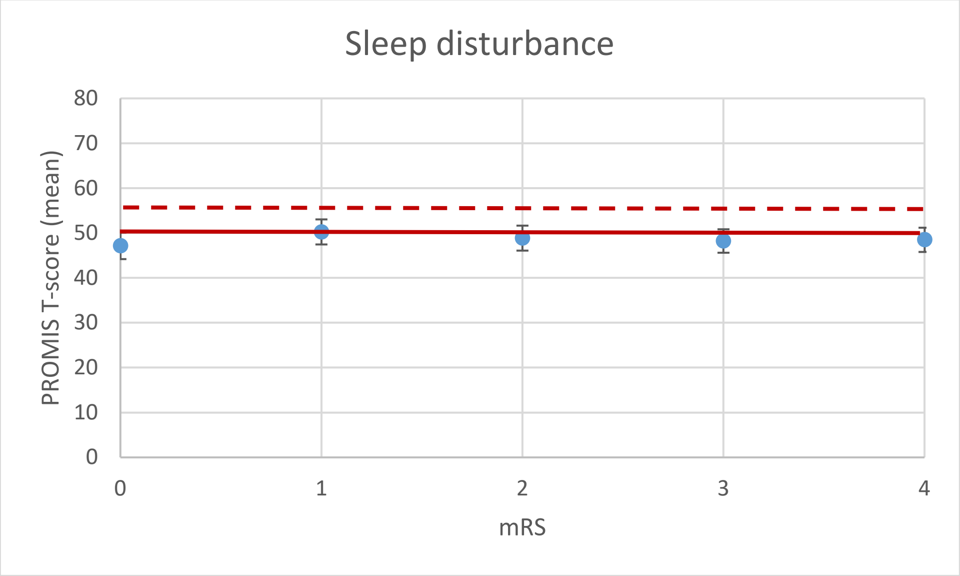

Supplementary figure 5 One dot represents the mean T-score; whiskers are the standard error of the T-score. The T-scores for mRS 0, 1, 2, 3 and 4 are respectively: 47.14 ± 2.96; 50.24 ± 2.79; 48.86 ± 2.78; 48.22 ± 2.61; 48.48 ± 2.70. The solid horizontal line represents the mean of the general population, the dotted horizontal line represents the score that is meaningfully worse than the general population. A higher T-score represents worse patient-reported health.

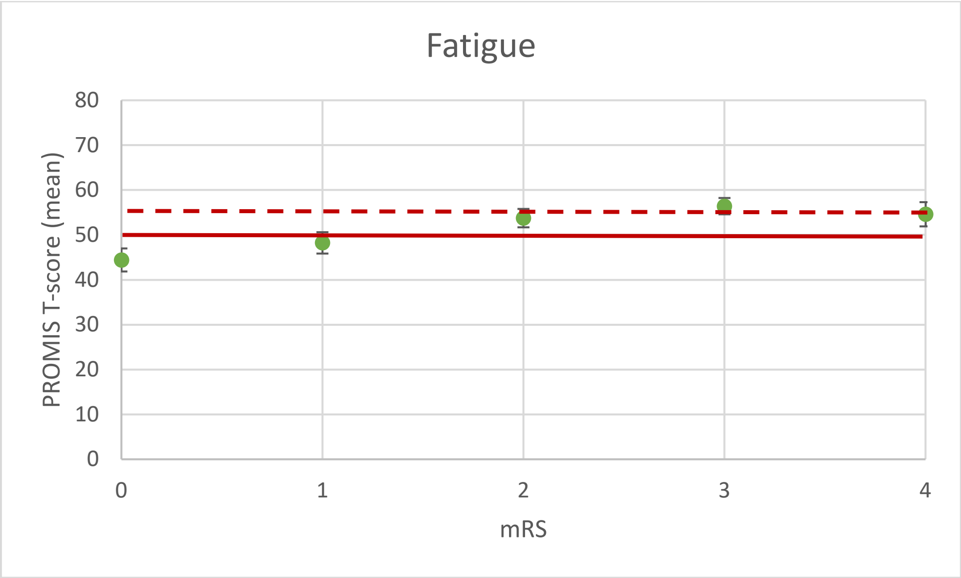

Supplementary figure 6 One dot represents the mean T-score; whiskers are the standard error of the T-score. The T-scores for mRS 0, 1, 2, 3 and 4 are respectively: 44.42 ± 2.57; 48.23 ± 2.38; 53.74 ± 2.05; 56.41 ± 1.82; 54.60 ± 2.70. The solid horizontal line represents the mean of the general population, the dotted horizontal line represents the score that is meaningfully worse than the general population. A higher T-score represents worse patient-reported health.

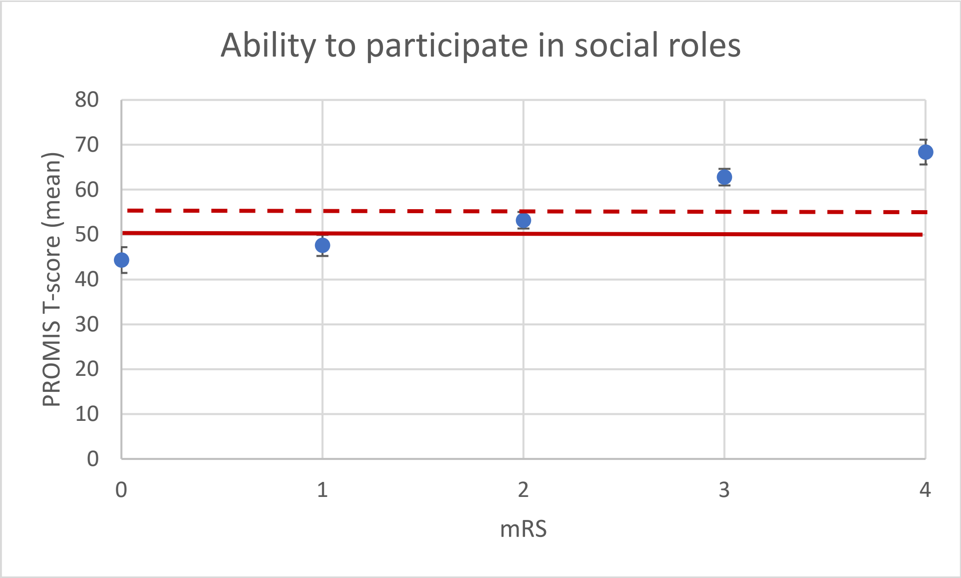

Supplementary figure 7 One dot represents the mean T-score; whiskers are the standard error of the T-score. The T-scores for mRS 0, 1, 2, 3 and 4 are respectively: 44.32 ± 2.87; 47.58 ± 2.35; 53.19 ± 1.85; 62.79 ± 1.85; 68.38 ± 2.76. The solid horizontal line represents the mean of the general population, the dotted horizontal line represents the score that is meaningfully worse than the general population. A higher T-score represents worse patient-reported health.

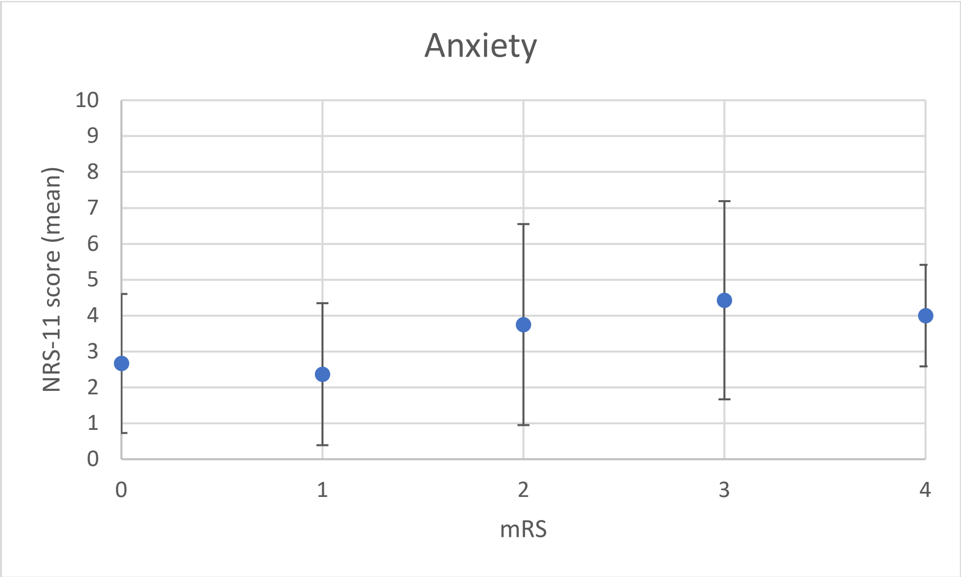

Supplementary figure 8 One dot represents the mean score on the scale of zero to ten; whiskers are the standard error of the mean score. The mean scores for mRS 0, 1, 2, 3 and 4 are respectively: 2.67 ± 1.94; 2.37 ± 1.98; 3.75 ± 2.80; 4.43 ± 2.76; 4.00 ± 1.41. A higher NRS-11 score represents worse patient-reported health.

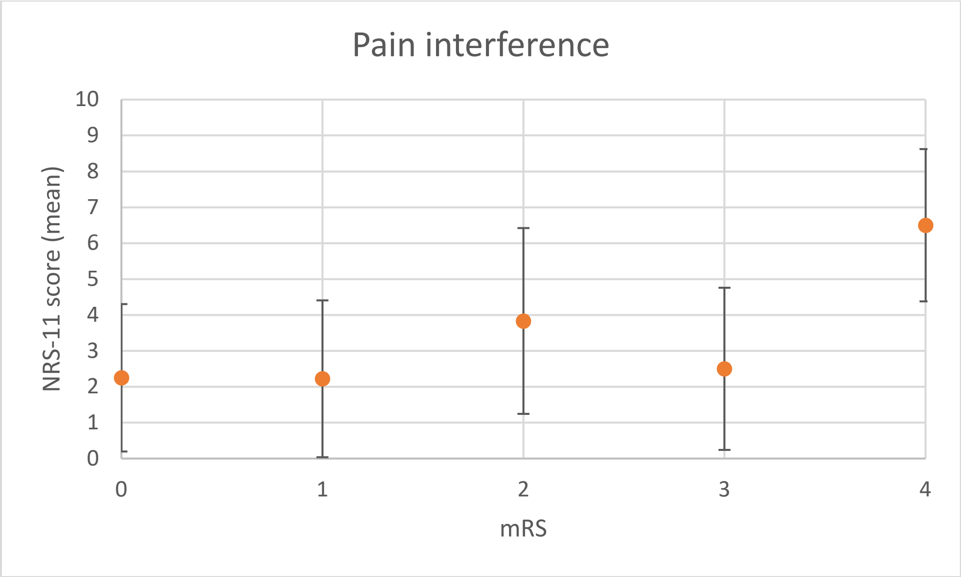

Supplementary figure 9 One dot represents the mean score on the scale of zero to ten; whiskers are the standard error of the mean score. The mean scores for mRS 0, 1, 2, 3 and 4 are respectively: 2.25 ± 2.05; 2.22 ± 2.18; 3.83 ± 2.59; 2.50 ± 2.26; 6.50 ± 2.12. A higher NRS-11 score represents worse patient-reported health.

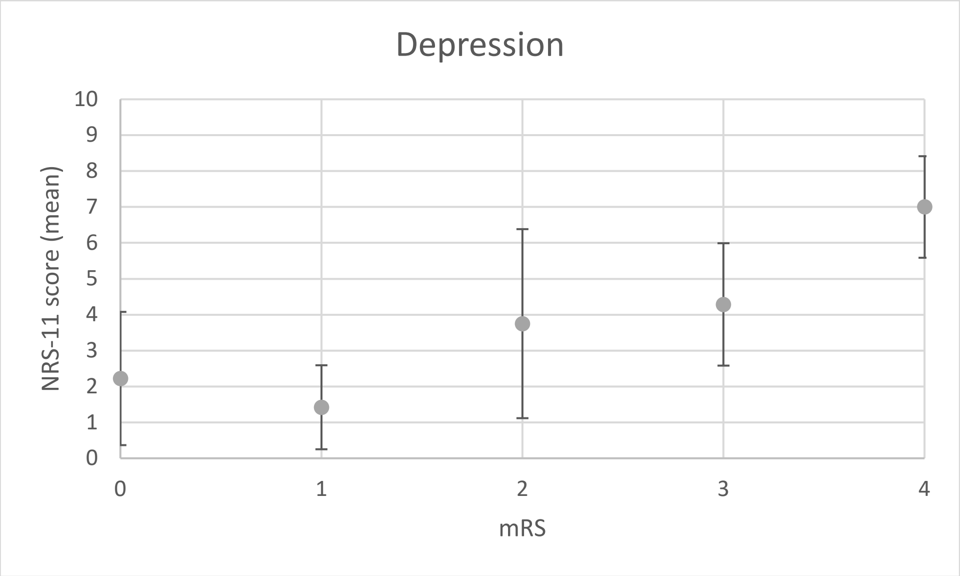

Supplementary figure 10 One dot represents the mean score on the scale of zero to ten; whiskers are the standard error of the mean score. The mean scores for mRS 0, 1, 2, 3 and 4 are respectively: 2.22 ± 1.86; 1.42 ± 1.17; 3.75 ± 2.63; 4.29 ± 1.70; 7.00 ± 1.41. A higher NRS-11 score represents worse patient-reported health.

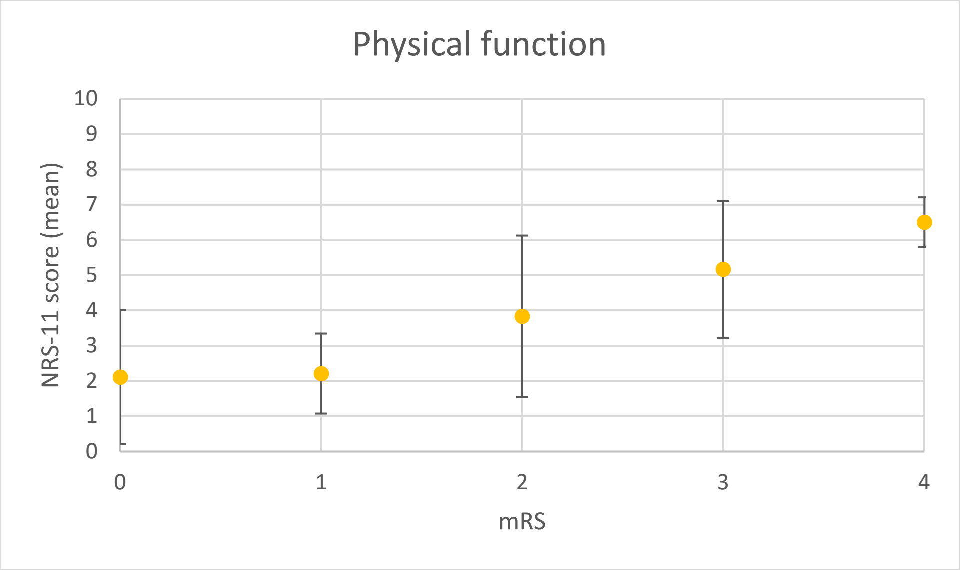

Supplementary figure 11 One dot represents the mean score on the scale of zero to ten; whiskers are the standard error of the mean score. The mean scores for mRS 0, 1, 2, 3 and 4 are respectively: 2.11 ± 1.90; 2.21 ± 1.13; 3.83 ± 2.29; 5.17 ± 1.94; 6.50 ± 0.71. A higher NRS-11 score represents worse patient-reported health.

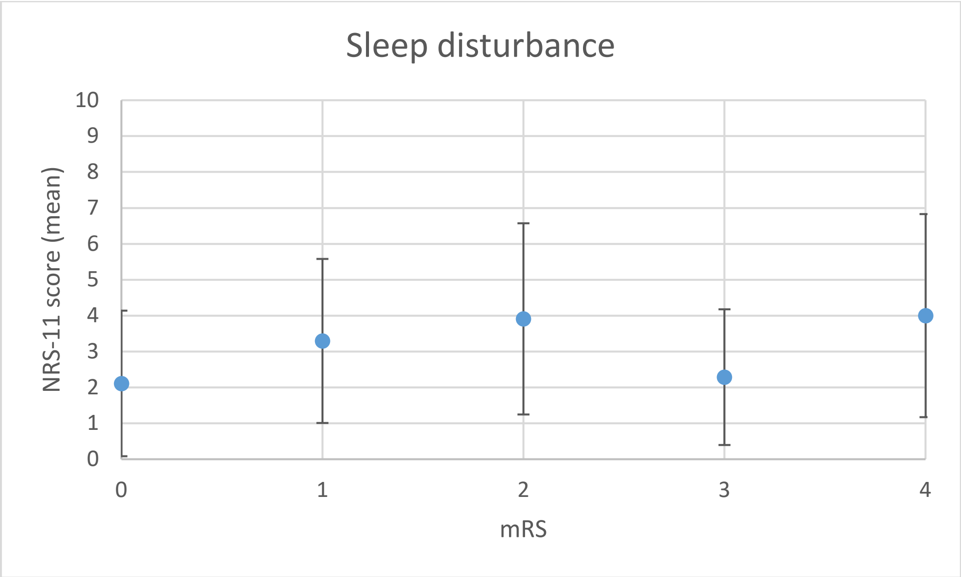

Supplementary figure 12 One dot represents the mean score on the scale of zero to ten; whiskers are the standard error of the mean score. The mean scores for mRS 0, 1, 2, 3 and 4 are respectively: 2.11 ± 2.03; 3.29 ± 2.28; 3.91 ± 2.66; 2.29 ± 1.89; 4.00 ± 2.83. A higher NRS-11 score represents worse patient-reported health.

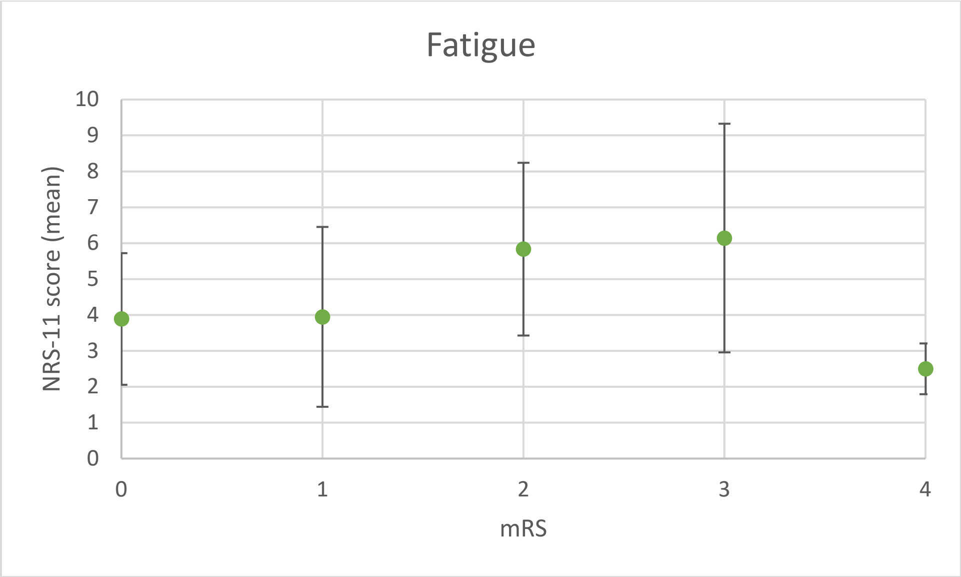

Supplementary figure 13 One dot represents the mean score on the scale of zero to ten; whiskers are the standard error of the mean score. The mean scores for mRS 0, 1, 2, 3 and 4 are respectively: 3.89 ± 1.83; 3.95 ± 2.50; 5.83 ± 2.41; 6.14 ± 3.18; 2.5 ± 0.71. A higher NRS-11 score represents worse patient-reported health.

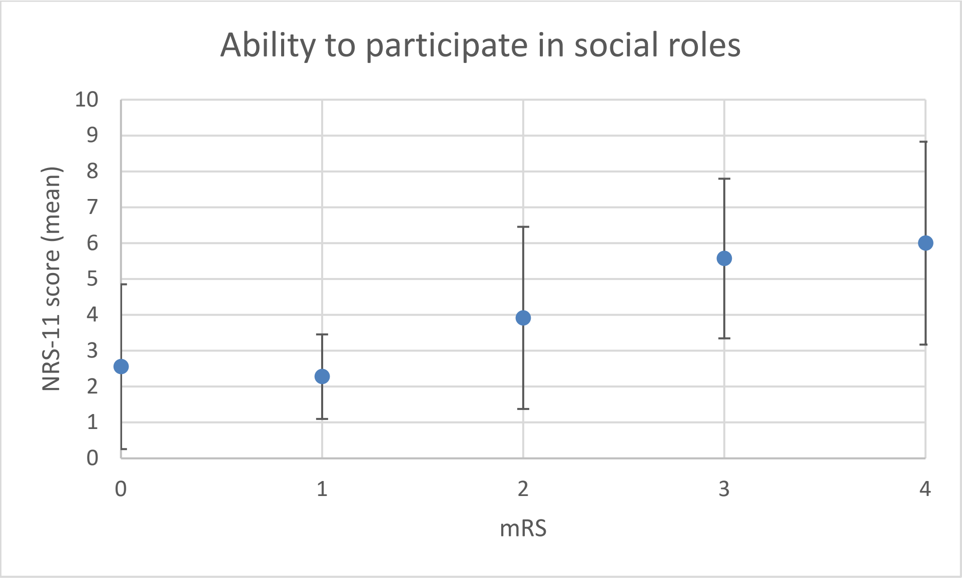

Supplementary figure 14 One dot represents the mean score on the scale of zero to ten; whiskers are the standard error of the mean score. The mean scores for mRS 0, 1, 2, 3 and 4 are respectively: 2.56 ± 2.30; 2.28 ± 1.18; 3.92 ± 2.54; 5.57 ± 2.23; 6.00 ± 2.83. A higher NRS-11 score represents worse patient-reported health.

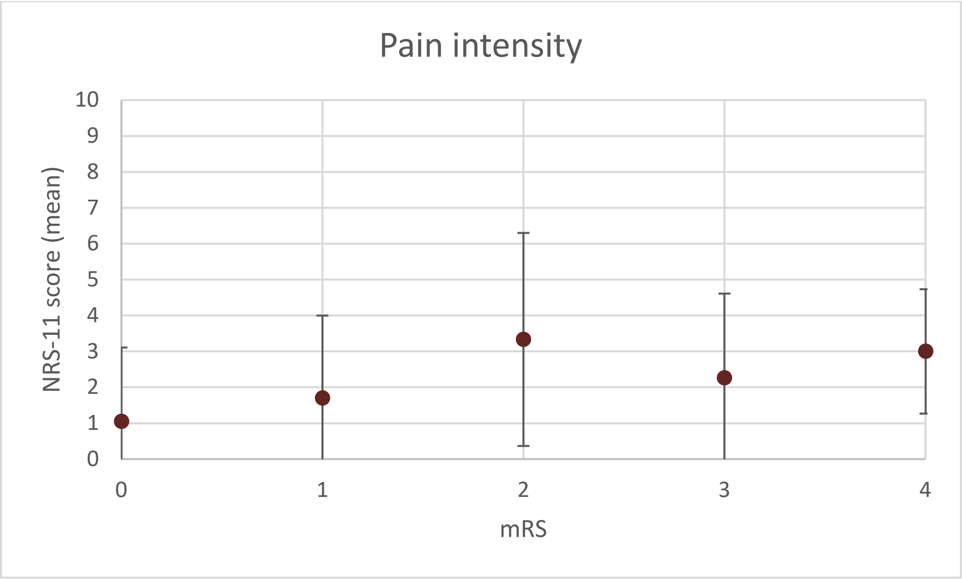

Supplementary figure 15 One dot represents the mean score on the scale of zero to ten; whiskers are the standard error of the mean score. The mean scores for mRS 0, 1, 2, 3 and 4 are respectively: 1.05 ± 2.06; 1.70 ± 2.30; 3.33 ± 2.97; 2.27 ± 2.34; 3.00 ± 1.73. A higher NRS-11 score represents worse patient-reported health.
